# Broad-Spectrum Antibiotic Regimen Affects Survival in Patients Receiving Nivolumab for Non-Small Cell Lung Cancer

**DOI:** 10.3390/ph14050445

**Published:** 2021-05-08

**Authors:** Min Jung Geum, Chungsoo Kim, Ji Eun Kang, Jae Hee Choi, Jae Song Kim, Eun Sun Son, Sun Min Lim, Sandy Jeong Rhie

**Affiliations:** 1 The Graduate School of Pharmaceutical Sciences, Ewha Womans University, Seoul 03760, Korea; MJGEUM@yuhs.ac; 2 Department of Pharmacy, Severance Hospital, Yonsei University Health System, Seoul 03722, Korea; CHH@yuhs.ac (J.S.K.); SESPHARM@yuhs.ac (E.S.S.); 3 Department of Biomedical Sciences, Ajou University Graduate School of Medicine, Suwon 16499, Korea; ted9219@ajou.ac.kr; 4 College of Pharmacy, Ewha Womans University, Seoul 03760, Korea; jjadu@nmc.or.kr; 5 Department of Pharmacy, National Medical Center, Seoul 04564, Korea; 6 Division of Life and Pharmaceutical Sciences, Ewha Womans University, Seoul 03760, Korea; 20110700@kuh.ac.kr; 7 Department of Pharmacy, Konkuk University Medical Center, Seoul 05030, Korea; 8 Division of Medical Oncology, Department of Internal Medicine, Yonsei University College of Medicine, Yonsei Cancer Center, Seoul 03722, Korea; LIMLOVE2008@yuhs.ac

**Keywords:** antibiotic-induced dysbiosis, days of therapy, defined daily dose, non-small cell lung cancer, nivolumab, overall survival

## Abstract

Antibiotic-induced dysbiosis may affect the efficacy of immune checkpoint inhibitors. We investigated the impact of antibiotics on the clinical outcomes of nivolumab in patients with non-small cell lung cancer (NSCLC). Patients who received nivolumab for NSCLC between July 2015 and June 2018 and who were followed up until June 2020 were included in a retrospective cohort analysis. Of 140 eligible patients, 70 were on antibiotics. Overall survival (OS) was shorter in patients on antibiotics (ABX) compared to those not on antibiotics (NoABX) (*p* = 0.014). OS was negatively associated with piperacillin/tazobactam (PTZ) (HR = 3.31, 95% CI: 1.77–6.18), days of therapy (DOT) ≥ 2 weeks (HR = 2.56, 95% CI: 1.30–5.22) and DOT of PTZ. The defined daily dose (DDD) in PTZ (r = 0.27) and glycopeptides (r = 0.21) showed weak correlations with mortality. There was no difference in progression-free survival (PFS) between ABX and NoABX; however, PFS was negatively associated with the antibiotic class PTZ and DOT of PTZ. Therefore, the use of a broad-spectrum antibiotic, such as PTZ, the long-term use of antibiotics more than 2 weeks in total and the large amount of defined daily dose of specific antibiotics were associated with decreased survival in patients receiving nivolumab for NSCLC.

## 1. Introduction

Nivolumab is an IgG4 anti-programmed cell death protein-1 (PD-1) monoclonal antibody. It binds to PD-1 on the surface of T cells and blocks PD-1-mediated signaling to help restore the body’s antitumor immunity [1]. The immune checkpoint inhibitors (ICIs) such as nivolumab, pembrolizumab and atezolizumab have shown significant clinical efficacy in treating advanced-stage non-small cell lung cancer (NSCLC) [2]. In Korea, nivolumab is an approved second-line therapy for NSCLC regardless of PD-L1 (programmed death ligand-1) expression [2,3]. It has been widely prescribed due to its vast clinical use and is easily reimbursed by the Korean National Health Insurance Services (NHIS) [4].

Some patients with advanced cancer have shown dramatic therapeutic responses to ICIs after other therapies have failed. However, other patients have partial or no response to anti-PD-1 or PD-L1 therapy [5,6,7]. Emergent research suggests that the gut microbiota might affect ICI response through immunoregulation [8,9,10,11,12]. Thus, disrupting the gut microbiota with antibiotic use, antibiotic-induced dysbiosis, has been implicated in the failure of immunotherapy within a variety of chronic inflammatory disorders. This indirect tumor-affecting activity is likely responsible for the decreased anti-cancer potential of anti-PD-1 antibodies in tumor-bearing mice [8].

Clinical studies of people with malignancies have shown that the use of broad-spectrum antibiotics at the beginning of immunotherapy is deleterious to patient response and survival due to severely reduced bacterial diversity and decreased intestinal flora function [8,9]. A retrospective cohort study by Derosa et al. showed that the administration of antibiotics within one month before the beginning of immunotherapy treatment negatively impacted the clinical response rate and survival of patients with metastatic renal cell carcinoma and NSCLC [6]. Routy et al. also reported worse progression-free survival (PFS) and overall survival (OS) in patients receiving antibiotics up to two months before or one month after the first immunotherapy compared with the group not receiving antibiotics in a large cohort of people with NSCLC, renal or urothelial carcinomas [8].

However, antibiotics are necessary to eradicate pathogens and prevent infection in immunocompromised patients with cancer [13,14]. However, there is no research in this population that clearly examines the optimal antibiotic type and dosage for treating infections and enhancing nivolumab treatment efficacy [15,16,17,18].

We aimed to investigate the effects of antibiotic use on the clinical outcomes in patients who received nivolumab monotherapy for advanced NSCLC and any associations with antibiotic class, exposure and dosage.

## 2. Results

### 2.1. Patient Characteristics and the Antibiotics Used

A total of 260 advanced NSCLC patients were treated with nivolumab monotherapy. We excluded eight patients from this study either because they died from pneumonia or nivolumab therapy was discontinued due to infections. A 1-to-1 propensity score matching was performed on 252 patients, and as a result, 140 patients were the eligible subjects of this study. 70 patients out of 140 eligible subjects were on antibiotics.

Baseline characteristics between the two groups were similar, and 71.4% of the participants were men. The mean age at first nivolumab use was 62.5 years (standard deviation, [SD], 11.1 years). The most common histological type was adenocarcinoma (70.0%, *n* = 98), and 76.4% (*n* = 107) of the participants had stage 4 NSCLC. Most had an ECOG performance score of 0 (97.1%, *n* = 136). The number of patients by PD-L1 expression was as follows: ≥50%, 36 (25.7%); 10% to <50%, 38 (27.1%); and <10%, 66 (47.1%) (Table 1). No patients were infected with *Clostridium difficile* or were prescribed probiotics.

The median duration of nivolumab monotherapy was 49 days and the median duration of follow-up was 31.3 months. The most common reason for discontinuation of nivolumab monotherapy was to change to another chemotherapy regimen. During the follow-up period, of the 70 patients treated with antibiotics (ABX), 28 patients died and 26 patients had disease progression. Of the other 70 patients who did not receive antibiotics (NoABX), 12 patients died and 39 patients had disease progression. The cause of death of enrolled patients who died during follow-up period was NSCLC.

**Figure 1:**
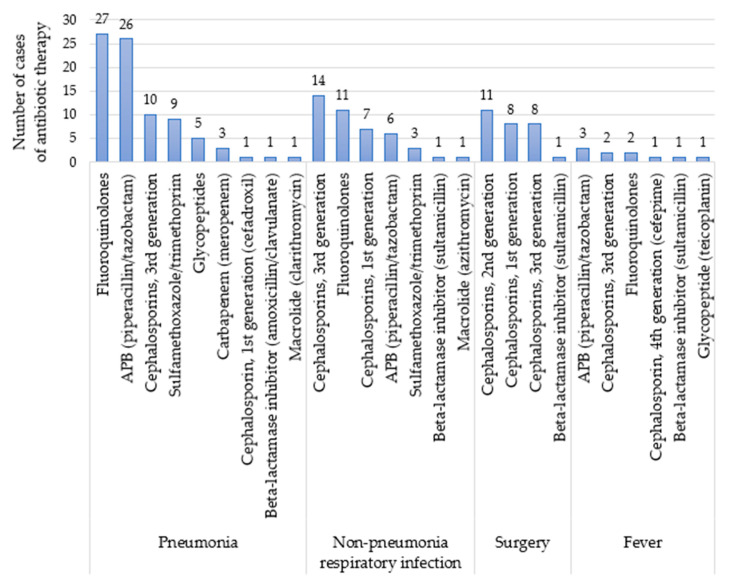
Antibiotic use by class for the four most frequent conditions reported. Abbreviation: APB, anti-pseudomonal beta-lactamase inhibitor.

Antibiotics of 14 different classes were used in the ABX group. The most common indication for antibiotic therapy was pneumonia. The most frequently administered antibiotics were levofloxacin (a fluoroquinolone) and piperacillin/tazobactam (PTZ; an anti-pseudomonal beta-lactamase inhibitor) (Figure 1). Other antibiotics used in this study were anti-tuberculosis drugs (ethambutol, isoniazid, pyrazinamide and rifampicin), beta-lactamase inhibitors (sultamicillin and amoxicillin/clavulanate), carbapenem (meropenem), first-generation cephalosporins (cefadroxil and cefazolin), second-generation cephalosporins (cefaclor, cefotetan and cefprozil), third-generation cephalosporins (ceftriaxone, cefpodoxime, cefditoren and cefixime), fourth-generation cephalosporin (cefepime), fluoroquinolones (ciprofloxacin and moxifloxacin), glycopeptides (teicoplanin and vancomycin), imidazole (metronidazole), macrolides (azithromycin and clarithromycin), sulfamethoxazole/trimethoprim and tetracycline (doxycycline). All antibiotics included in this study were administered systemically. The median duration of each antibiotic treatment was seven days.

### 2.2. OS and PFS

**Figure 2:**
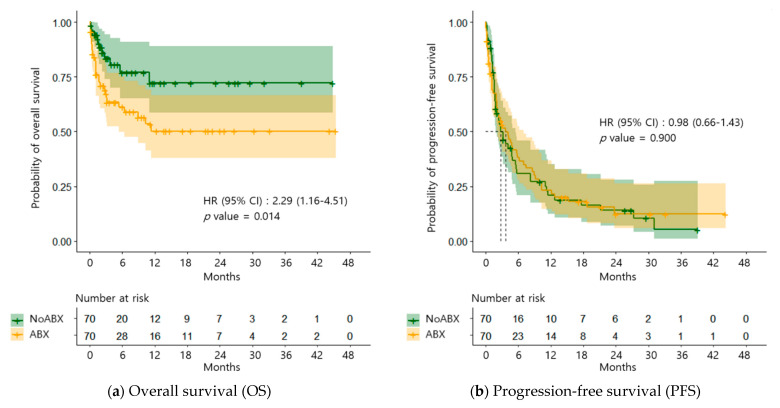
Kaplan-Meier survival plots for overall survival (**a**) and progression-free survival (**b**) in both patients who treated with nivolumab for non-small cell lung cancer with antibiotics (ABX group, *n* = 70) or without antibiotics (NoABX group, *n* = 70).

The OS was significantly lower in the ABX group compared to that of the NoABX group (HR = 2.29, 95% CI: 1.16–4.51, *p* = 0.014) (Figure 2a). However, the PFS did not differ significantly between the two groups (HR = 0.98, 95% CI: 0.66–1.43, *p* = 0.900) (Figure 2b).

### 2.3. Associated Factors with OS and PFS

#### 2.3.1. Antibiotic Class

PTZ was significantly associated with a short OS (HR = 3.31, 95% CI: 1.77–6.18, *p* < 0.001) and short PFS (HR = 3.40, 95% CI: 1.82–6.34, *p* < 0.001) (Table 2).

#### 2.3.2. Antibiotic Days of Therapy (DOT)

**Figure 3:**
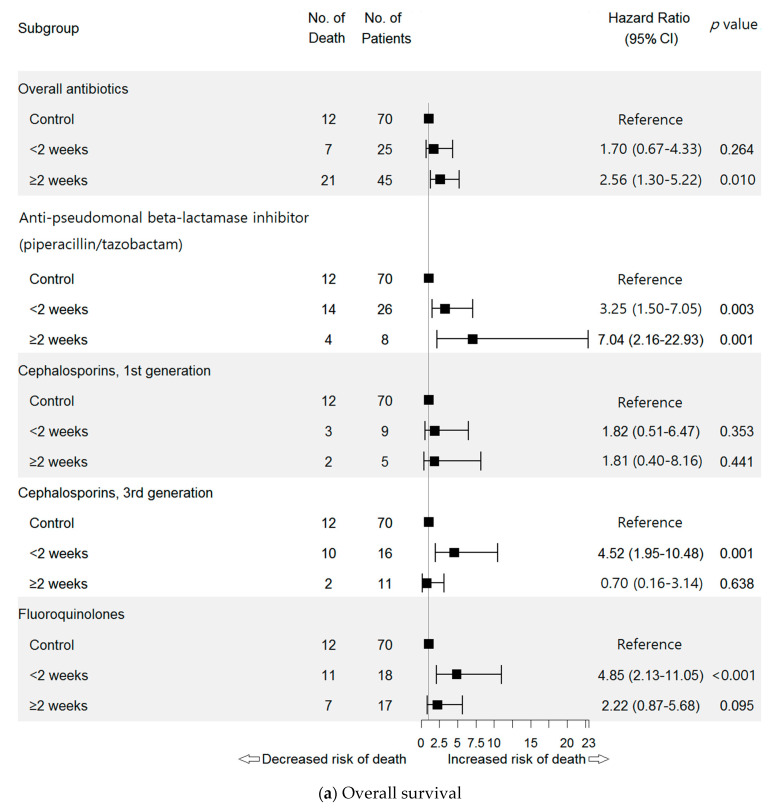

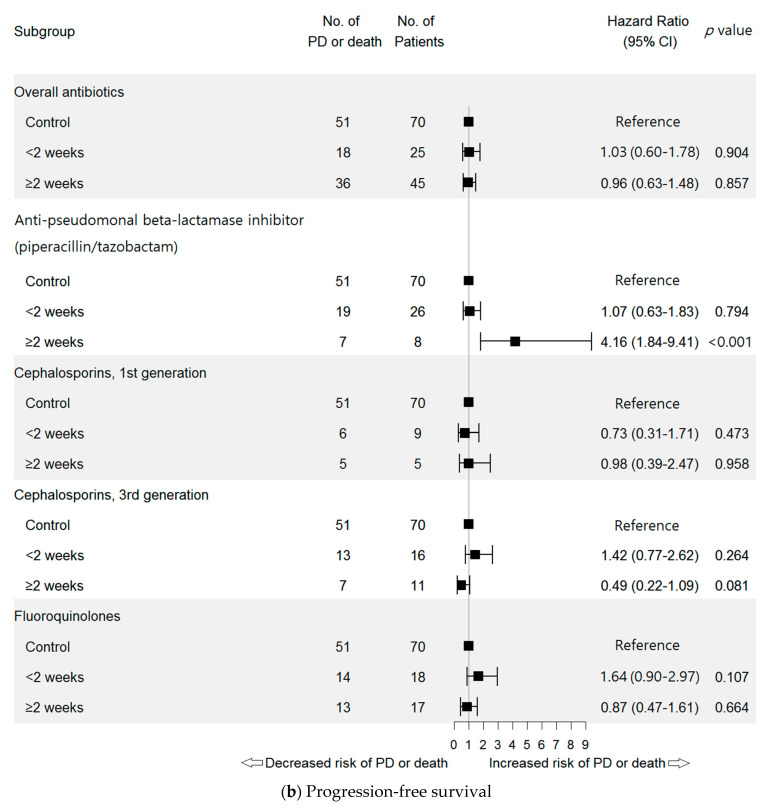
Hazard ratios for overall survival (**a**) and progression-free survival (**b**) by antibiotic days of therapy (DOT). Each control refers to the patients who have not used antibiotics (NoABX). Anti-tuberculosis medications, beta-lactamase inhibitors, carbapenem (meropenem), second-generation cephalosporins, fourth-generation cephalosporin (cefepime), glycopeptides, imidazole (metronidazole), macrolides, sulfamethoxazole/trimethoprim and tetracycline (doxycycline) were not included in this forest plot due to small sample sizes (<5). Abbreviations: No., number; PD, progression of disease.

A Cox proportional hazards-model identified the DOT of overall antibiotics as negative factor associated with OS (for ≥2 weeks; HR = 2.56, 95% CI: 1.30–5.22, *p* = 0.010) (Figure 3a).

DOT of PTZ treatment was negatively associated with OS (for < 2 weeks: HR = 3.25, 95% CI: 1.50–7.05, *p* = 0.003; for ≥2 weeks: HR = 7.04, 95% CI: 2.16–22.93, *p* = 0.001) (Figure 3a). The negative association of PTZ was also found with PFS (for ≥2 weeks: HR = 4.16, 95% CI: 1.84–9.41, *p* < 0.001) (Figure 3b).

Moreover, the DOT of third-generation cephalosporins was negatively associated with OS (<2 weeks: HR = 4.52, 95% CI: 1.95–10.48, *p* = 0.001), as was fluoroquinolone use (<2 weeks use: HR = 4.85, 95% CI: 2.13–11.05, *p* < 0.001) (Figure 3a).

#### 2.3.3. Defined Daily Dose (DDD) of Antibiotics

By June 2020 (the end of data obtained), there were 100 surviving patients and 40 deceased patients. The mean antibiotic consumption in DDD was 13.1 in the surviving patients and 16.4 in the deceased patients. DDD in PTZ (r = 0.27, *p* = 0.001) and glycopeptides (r = 0.21, *p* = 0.012) showed a weak positive correlation with mortality (Table 3). There were 105 patients with tumor progression or who died and 35 patients with no tumor progression. The mean antibiotic consumption in DDD was 15.7 in the progression-free surviving patients and 16.6 in the progressed or deceased patients. There was no correlation between PFS and antibiotic DDD (Table 3).

## 3. Discussion

This study investigated the impact of antibiotics on the survival rates of NSCLC patients treated with nivolumab as a retrospective cohort study. We aimed to analyze associations with the class, duration and dose of the antibiotics and the clinical outcome of nivolumab. We excluded possible confounding variables with thorough exclusion criteria for patients with active infections, propensity score matching and included patient with nivolumab monotherapy for NSCLC. Moreover, we used two measuring methods, the DOT and the number of DDD, that are most objective and comparable representation of the duration and amount of antibiotics used. Antibiotic use was negatively associated with survival rate in patients receiving nivolumab for NSCLC. Specifically, our findings confirm that the use of broad-spectrum antibiotics such as PTZ, the long-term use of antibiotics for more than 2 weeks in total, and the large amount of specific antibiotics consumption were associated with lower survival rates in patients receiving nivolumab for NSCLC.

In a systematic review by Elkrief et al., 11 of 12 retrospective studies reported negative effects of antibiotic use on the clinical outcomes of patients using ICIs for various cancers including NSCLC, renal cell carcinoma and melanoma [16]. The authors believed that dysbiosis caused by antibiotic use may adversely affect ICI effectiveness. Our results confirmed that antibiotic use significantly worsened the OS in NSCLC patients receiving nivolumab [6,16,19,20,21,22]. However, no significant association between antibiotic use and PFS was identified, although a negative trend was observed.

We selected nivolumab for our study and focused on one type of advanced NSCLC. We wanted to investigate the clinical outcomes of frequently used antibiotics in this population.

Several studies have reported antibiotic use and its effects on the gut microbiome composition and the clinical impact of patients on ICIs [15,16]. Previously, the exposure period of eligible patients to systemic antibiotics was mostly from one to two months before or one month after initiation of ICIs [16,23]. When antibiotic-induced dysbiosis occurs, the intestinal microbiota composition may be disrupted for several weeks [24]. Moreover, dysbiosis could continue as long as antibiotics are administered during ICI treatment. Therefore, we set the critical timeframe of an “antibiotic window” from one month before nivolumab initiation to the end of nivolumab therapy, similar to a study by Pinato et al. [25].

PTZ is a preferred broad-spectrum antibiotic for patients at high risk of multidrug-resistant pathogens or when *Pseudomonas aeruginosa* is suspected. In this study, we found that it was primarily used for pneumonia. The use of PTZ was profoundly associated with OS. PTZ has a significant effect on the gut microbiota, and some studies indicate a substantial decrease in anaerobic commensals four to eight days after using it [26,27,28]. Specifically, PTZ reduces the population profile of *Bifidobacterium*, *Eubacterium* and *Lactobacillus* in human gut microbiota [26,29]. These are known major gut microbiome strains in ICI responders [30]. *Bifidobacterium* is especially abundant in Chinese individuals who respond to anti-PD-1 immunotherapy against NSCLC [31].

Responders and non-responders to ICIs have a different intestinal microbiome, and there is an important link between commensal microbial composition and clinical response of ICIs [30]. Therefore, PTZ could shift the composition of gut bacteria and reduce favorable strains in NSCLC patients receiving nivolumab.

We found that OS was associated with the class, duration, and dose of antibiotics, which was similar to previous studies [17,32]. In our analysis, we calculated DOT to express the duration of antibiotic therapy by the sum of cumulative exposed days between one month before and the end of nivolumab treatment. We also applied DDD to objectively compare the usage of different antibiotics by calculating a fixed unit of measurement of the average maintenance dose per day for a drug. Both DOT and DDD methods are representative measurement methods standardized for comparing antibiotic consumption.

A statistically significant correlation was observed between the duration of PTZ consumption and mortality risk in our patients treated with nivolumab. Other studies have highlighted the importance of the duration of antibiotic use in patients on ICIs, as longer periods of antibiotic use may affect patient prognosis [16,33]. Evidence suggests that a shorter duration of antibiotic use can reduce adverse effects [34]. Furthermore, systematic reviews have found that clinical outcomes were similar between short and long antibiotic courses for many common infections [34]. A better dosing strategy can improve clinical safety and efficacy outcomes when administering PTZ.

Levofloxacin was a fluoroquinolone prescribed to our patients. According to Ziegler et al., there was no association between DOT of levofloxacin and gut microbiome diversity [35]. However, we observed an association between DOT of fluoroquinolones (at <two weeks) and overall survival. Levofloxacin affects a greater proportion of *Ruminococcaceae* differently than broad-spectrum beta-lactams [35]. *Ruminococcaceae* enhances the PD-1 inhibition effect and effector T cell functioning [30,36,37]. While the research is mixed [31], the administration of levofloxacin may induce a shift in the gut microbiome composition, altering the effect of nivolumab, a PD-1 inhibitor.

Among the third-generation cephalosporins, cefditoren, cefixime, cefpodoxime and ceftriaxone were prescribed in this study. Each drug has a slightly different effect on intestinal bacteria, but they tend to decrease *Escherichia* and increase *Enterococcus* [17]. *Enterococcus* is a major bacterium in the gut microbiome of ICI responders, which increases T cell response and enhances the PD-1 blockade effect [30,37,38]. In this study, the third-generation cephalosporins only influenced OS early in cephalosporin treatment. However, longitudinal analysis with direct measurements of the gut microbiome warrants future study.

This study has several limitations. The infection severity indicated that the antibiotics were not included in the analysis and that the antibiotics were not randomly allocated as part of the retrospective study design. However, to reduce selection bias, we excluded patients who died from infection and those who discontinued nivolumab therapy due to infection. Moreover, we conducted propensity matching to remove baseline discrepancies such as ECOG and cancer stage. This was a retrospective study at a single center involving a small number of patients. We had stringent exclusion criteria and used a matching process. Our number of subjects was comparable to previous human studies. Unfortunately, the antibiotic combinations were not considered. Further, the subjects’ gut microbiota samples were not assessed due to the retrospective nature of the study. Lastly, we were unable to incorporate smoking status, EGFR mutation and medication history. We believe those variables would not make a substantial difference in the analysis as patients who meet the criteria were eligible for nivolumab treatment.

Despite the above limitations, our study is different from previous studies in three ways. First, we analyzed the clinical outcomes of nivolumab in NSCLC patients by OS, PFS and the antibiotic class, duration and amount. Second, excluded potential confounding variables before analysis. Finally, we introduced two measurements (DOT and DDD) to allow for an objective comparison between the ABX and NoABX groups.

Different from the previous studies, we tried to determine which characteristics of the antibiotic therapy were related to the clinical outcome of the ICI treatment. In this study, we found that the use of a broad-spectrum antibiotic, such as PTZ, the long-term use of antibiotics more than 2 weeks in total, and the increased consumption of defined daily dose of specific antibiotics were negatively associated with overall survival. So far, there have been existing evidences that antibiotics overall have a negative impact on the effect of ICI [16], without consider of specific management of antibiotic use in clinical settings. Through the result of this study, we suggest specific and applicable rationale for the use of antibiotics during nivolumab therapy for NSCLC.

## 4. Materials and Methods

### 4.1. Patients

Patients with NSCLC who received nivolumab monotherapy at Yonsei University Health System between July 2015 and June 2018 were eligible for this study. Patients who died from active infection or whose nivolumab treatment was discontinued due to infection were excluded. Patient demographics, pathology and prescription data, including age, sex, cancer stage, Eastern Cooperative Oncology Group (ECOG) performance status, histological type, PD-L1 expression, the number of prior treatment regimens and history of antibiotic use, were collected by a retrospective chart review.

Patients were divided into two groups. Patients administered systemic antibiotics within one month before the start of nivolumab or concurrently with nivolumab were defined as those treated with antibiotics (ABX). The ABX group was compared to those patients not administered systemic antibiotics (NoABX). Age was categorized as ≥65 or <65 years; the number of prior chemotherapy regimens was categorized as ≥3 or <3; and PD-L1 expression was categorized as high (≥50%), moderate (10–<50%) or low (<10%). Antibiotics were classified by the Access, Watch, Reserve (AWaRe) classification of antibiotics system by the World Health Organization (WHO) [39]. Censored date included a change in chemotherapy regimen, those lost to follow-up or transferred to other hospitals and treatment discontinuation due to financial problems.

The OS was defined as the time from nivolumab initiation to death from any cause or until the last follow-up in June 2020. PFS was defined as the time from nivolumab initiation to objectively evident tumor progression, death from any cause or the last follow-up, whichever occurred first. Tumor progression was determined using the Response Evaluation Criteria in Solid Tumors (RECIST), version 1.1 [40].

The days of therapy (DOT) were calculated as the sum of the number of days that a patient received an antibiotic from four weeks before the start of nivolumab therapy to the entire nivolumab monotherapy period, regardless of the given dose [41]. Therefore, the DOT is not a consecutive exposure, but a cumulative one.

The defined daily dose (DDD) was used to assess the amount of antibiotics. It refers to the assumed average maintenance dose per day for a drug used according to WHO indications in adults [42]. Thus, the number of DDD was defined as the total amount of antibiotic doses divided by the WHO DDD [43,44].

### 4.2. Statistical Analysis

To adjust for the baseline confounders, a 1-to-1 propensity score matching between groups was performed using the nearest-neighbor matching method with a caliper width of 0.2. Variables included in the propensity score matching algorithm included age, sex, cancer stage, ECOG performance status, histology, the number of prior treatment regimens and PD-L1 expression. Imputation of missing data was not allowed and patients with missing values were excluded from the analysis.

Baseline patient characteristics of the ABX and NoABX groups were summarized by descriptive statistics and compared using the Chi-squared test or Fisher’s exact test for categorical variables and t-test for continuous variables. The Kaplan-Meier estimator was used to assess the fraction of OS and PFS, and the log-rank test was used to compare the groups.

To identify the prognostic factors associated with OS and PFS in all patients, a univariable hazard ratio (unadjusted HRs) was calculated using the Cox regression model. A multivariable Cox regression was then performed with all of the variables with *p* < 0.2 in the univariable analysis. The Cox regression models were selected using backward stepwise procedures to select covariates for the final model. We obtained adjusted HRs and 95% confidence intervals (CIs) for OS and PFS using the multivariable Cox regression analysis.

A subgroup analysis was performed using Cox proportional hazards regression to determine the effects of treatment duration (DOT) on OS and PFS by each antibiotic class. Proportional hazards assumptions were evaluated by assessing Schoenfelds residuals. Point-biserial correlation analyses were performed to evaluate the correlation between clinical outcomes and amount of antibiotics (DDD). Through the subgroup analysis, HRs, 95% CIs and p-values for OS and PFS were obtained for each subgroup. Statistical tests were 2-sided, and a *p*-value < 0.05 was considered statistically significant. All statistical analyses were performed using R version 4.0.2 (R Foundation for Statistical Computing, Vienna, Austria).

## 5. Conclusions

Our study confirms that antibiotic use negatively affects overall survival in NSCLC patients treated with nivolumab, possibly due to antibiotic-induced dysbiosis. This study showed that the use of broad-spectrum antibiotics such as PTZ, longer antibiotic use more than 2 weeks and an increased defined daily dose of PTZ or glycopeptides were negatively associated with overall survival in patients receiving nivolumab for NSCLC.

Therefore, careful selection and optimal dosing of antibiotics may effectively manage nivolumab treatment for advanced NSCLC. Protocols and manuals for ICIs should be prepared for patients with clinically suspected or confirmed infections being treated with antibiotics.

## Figures and Tables

**Table 1:** Baseline patient characteristics matched by propensity score.

Variables	Total *n* = 140	NoABX *n* = 70	ABX *n* = 70	*p*-Value
Age				
Mean ± SD	62.5 ± 11.1	63.2 ± 9.4	61.7 ± 12.6	0.693
<65 years, *n* (%)	78 (55.7)	40 (57.1)	38 (54.3)	0.865
≥65 years, *n* (%)	62 (44.3)	30 (42.9)	32 (45.7)	
Sex, *n* (%)				
Female	40 (28.6)	20 (28.6)	20 (28.6)	1.000
Male	100 (71.4)	50 (71.4)	50 (71.4)	
Stage, *n* (%)				
2	3 (2.1)	1 (1.4)	2 (2.9)	0.634
3	18 (12.9)	11 (15.7)	7 (10.0)	
4	107 (76.4)	51 (72.9)	56 (80.0)	
Recurrence	12 (8.6)	7 (10.0)	5 (7.1)	
ECOG performance status, *n* (%)				
0	136 (97.1)	69 (98.6)	67 (95.7)	0.620
1	4 (2.9)	1 (1.4)	3 (4.3)	
Tumor subtype, *n* (%)				
Adenocarcinoma	98 (70.0)	50 (71.4)	48 (68.6)	0.854
Squamous cell carcinoma	42 (30.0)	20 (28.6)	22 (31.4)	
Number of regimens before nivolumab				
<3, *n* (%)	80 (57.1)	41 (58.6)	39 (55.7)	0.864
≥3, *n* (%)	60 (42.9)	29 (41.4)	31 (44.3)	
PD-L1 expression, *n* (%)				
High (≥50%)	36 (25.7)	19 (27.1)	17 (24.3)	0.918
Moderate (≥10%, <50%)	38 (27.1)	19 (27.1)	19 (27.1)	
Low (<10%)	66 (47.1)	32 (45.7)	34 (48.6)	

**Table 2:** Univariable and multivariable analyses of overall survival and progression-free survival by antibiotic class.

Variables *	Overall Survival				Progression-Free Survival			
	Univariable Analysis		Multivariable Analysis		Univariable Analysis		Multivariable Analysis	
	HR (95% CI)	*p*-Value	HR (95% CI)	*p*-Value	HR (95% CI)	*p*-Value	HR (95% CI)	*p*-Value
Anti-pseudomonal beta-lactamase inhibitor (piperacillin/tazobactam)	3.31 (1.77–6.18)	<0.001	3.31 (1.77–6.18)	<0.001	1.43 (0.91–2.23)	0.119	3.40 (1.82–6.34)	<0.001
Beta-lactamase inhibitors	0.28 (0.04–2.11)	0.220			0.47 (0.19–1.16)	0.100	-	-
Cephalosporins, 1st generation	1.17 (0.46–2.98)	0.749			0.83 (0.44–1.54)	0.549		
Cephalosporins, 2nd generation	0.50 (0.12–2.09)	0.345			0.78 (0.39–1.56)	0.489		
Cephalosporins, 3rd generation	1.67 (0.85–3.28)	0.140	-	-	0.84 (0.52–1.37)	0.492		
Fluoroquinolones	2.88 (1.54–5.37)	<0.001	-	-	1.24 (0.80–1.92)	0.338		
Glycopeptides	3.22 (1.26–8.25)	0.015	-	-	1.40 (0.65–3.03)	0.391		
Sulfamethoxazole/ trimethoprim	2.24 (0.99–5.06)	0.053	-	-	1.08 (0.58–2.03)	0.802		

* Anti-tuberculosis medications, carbapenem (meropenem), fourth-generation cephalosporin (cefepime), imidazole (metronidazole), macrolides and tetracycline (doxycycline) were not included in this univariable and multivariable analysis due to small sample sizes (<5).

**Table 3:** Correlation between the defined daily dose (DDD) and overall survival and progression-free survival by antibiotic class.

Variables	Overall Survival				Progression-Free Survival			
	DDD, Mean ± SD		r	*p*-Value	DDD, Mean ± SD		r	*p*-Value
	Survival *n* = 100	Death *n* = 40			Progression- Free Survival *n* = 35	Progressed Disease or Death *n* = 105		
Overall antibiotics	13.1 ± 33.7	16.4 ± 33.3	0.15	0.068	15.7 ± 29.1	16.6 ± 34.7	0.01	0.896
Anti-pseudomonal beta-lactamase inhibitor (piperacillin/tazobactam)	1.7 ± 4.9	2.8 ± 6.5	0.27	0.001	2.2 ± 5.5	3.0 ± 6.8	0.05	0.576
Anti-tuberculosis medications	-	0.3 ± 3.9	0.13	0.114	-	0.3 ± 3.9	0.05	0.566
Beta-lactamase inhibitors	0.4 ± 2.7	0.3 ± 2.3	−0.06	0.514	0.1 ± 0.5	0.4 ± 2.7	0.05	0.544
Carbapenem (meropenem)	0.5 ± 4.6	0.5 ± 4.2	0.02	0.810	1.3 ± 7.8	0.2 ± 1.9	−0.11	0.196
Cephalosporins, 1st generation	0.7 ± 2.8	0.7 ± 2.8	0.01	0.891	0.2 ± 0.8	0.8 ± 3.1	0.09	0.280
Cephalosporins, 2nd generation	0.3 ± 1.3	0.3 ± 1.2	−0.04	0.608	0.0 ± 0.2	0.4 ± 1.3	0.13	0.134
Cephalosporins, 3rd generation	1.6 ± 4.7	1.7 ± 4.5	−0.05	0.595	2.3 ± 6.2	1.5 ± 3.8	−0.07	0.392
Cephalosporin, 4th generation (cefepime)	-	0.2 ± 2.0	0.13	0.114	-	0.2 ± 2.3	0.05	0.566
Fluoroquinolones	4.1 ± 11.0	4.9 ± 11.0	0.12	0.156	7.1 ± 15.0	4.2 ± 9.3	−0.11	0.181
Glycopeptides	0.1 ± 0.6	0.3 ± 1.6	0.21	0.012	0.1 ± 0.5	0.4 ± 1.8	0.08	0.335
Imidazole (metronidazole)	-	0.5 ± 5.9	0.13	0.114	-	0.7 ± 6.8	0.05	0.566
Macrolides	0.1 ± 1.0	0.3 ± 2.6	0.12	0.142	0.2 ± 1.4	0.4 ± 2.9	0.02	0.794
Sulfamethoxazole/trimethoprim	3.1 ± 23.0	3.0 ± 20.0	−0.01	0.918	0.9 ± 4.4	3.7 ± 22.9	0.06	0.481
Tetracycline (doxycycline)	0.8 ± 7.7	-	−0.05	0.529	2.2 ± 13.0	-	−0.15	0.083

## Data Availability

The data presented in this study are available on request from the corresponding author. The data are not publicly available due to privacy restrictions and patient confidentiality.
